# Opening Gated Communities and Neighborhood Accessibility Benefits: The Case of Seoul, Korea

**DOI:** 10.3390/ijerph18084255

**Published:** 2021-04-16

**Authors:** Omer Dogan, Jaewon Han, Sugie Lee

**Affiliations:** Department of Urban Planning and Engineering, Hanyang University, 222 Wangsimni-ro, Seongdong-gu, Seoul 04763, Korea; omerdogan1088@gmail.com (O.D.); tommorello@hanmail.net (J.H.)

**Keywords:** gated community, accessibility, permeability, apartment complex, network analysis, walkability

## Abstract

The level of spatial accessibility is directly related to how street networks are connected. Connected or so-called “permeable” network systems encourage walking, cycling, and riding public transit. Fast urbanization during the recent decades in the world metropolises has created separated urban areas. Gated-style apartment complexes have led this segregation more obviously with their inaccessible internal networks. Opening the internal network of apartment complexes and redesigning the pedestrian paths among apartment buildings will significantly mitigate these networks’ adverse effects on network permeability and increase spatial accessibility. This paper analyzes how such an opening design proposal for apartment complexes can change spatial accessibility using the case study of Mapo-gu, Seoul, Korea. It simulates three types of street networks and compares the results of accessibility in three conditions: (1) the internal networks of apartment complexes are not used by outsiders; (2) the internal networks of apartment complexes are open to outsiders with its existing entrances and path; and (3) the internal networks of sites are opened and redesigned by the Voronoi diagram method, which generates the optimal shortest path. An urban network analysis tool, Rhinoceros three-dimensional software, and Grashopper3D visual programming language have been used for the study results, which shows that a policy change in opening the intra-network of apartment complexes is likely to make the city more permeable. In addition, this study suggests extra modification on the pedestrian path for a higher level of accessibility in neighborhoods.

## 1. Introduction

Walking distance is an essential factor influencing whether or not people choose public transport, especially in areas of large residential sites and non-central suburbs [1,2]. Some studies found that it has the highest proportion among the factors influencing transit ridership [3]. In this sense, the characteristics of a built environment that increase the distances in walking routes to public transit should be well examined to make better policies. As an example of such characteristics, large apartment complexes are expected to increase walking distances to public transit.

The apartment is a representative type of housing in Korea. According to the Korean Population and Housing Census [4], more than half the country’s population live in apartments. An apartment has the characteristic of many households occupying a single building, which justifies the independence of individual families and private ownership of the shared space within the apartment complex. Individual households’ autonomy and the shared space for residents within the complex provide a pleasant and intimate living environment. Still, due to the lack of consideration for the apartment complex’s external environment, apartment sites have been criticized in that they promote a physical disconnection from their surrounding area to cause a closed community.

Another aspect of Korean apartment complexes is that they occupy larger areas than do examples in other countries. This increases the impact of disconnection [5]. The privatization of excessively occupied spaces weakens the sense of urban community and causes the disconnection of neighboring social networks, making the neighborhood’s social sustainability at risk. In addition, the large-scale apartment complexes degrade neighborhood environmental sustainability with disconnected open spaces. Studies show that the percentage of students walking to school has decreased from 49% in 1969 to only 13% in 2009 [6]. This result is related to both the change in transportation systems and the influence of urban forms, which hinder walking activity. In Dogan et al. [5], we focused on the impact of large-size apartment complex developments and their influence on walking environments in neighborhoods. We found that with the development of large-scale apartment complexes, the walking time to schools increased, causing a decline in walking rate and more automobile dependency.

The internal pedestrian paths of apartment complexes are mostly different from the typical surrounding neighborhood’s pedestrian networks by design. Apartment complexes have more irregular and ornamented paths that are shaded or lighted. Some may claim that this increases walking comfort, but such an irregular path causes unfamiliarity, because pedestrians from outside may feel more unaware of the place, which is a reason not to choose such a walking route. In addition, the isolated walking path of the apartment complex’s residents robs the building of a sense of community by reducing social contact and hindering the formation of relationships with neighbors. In addition, privatizing roads and public spaces in the complex, limiting cars and pedestrians’ inflow, and emphasizing security cause a massive segregation in the city center.

Internal streets of Chinese, South American, and Middle Eastern gated communities are segregated from surrounding street networks by gates and walls, whereas some apartment complexes in Korea do not hinder pedestrians from passing through the gates into their internal network. However, this does not mean a good connection between the neighborhood pedestrian network and the apartment complexes’ internal network. Although some local people may be familiar with apartment complexes’ internal street networks, many may not use pedestrian paths inside apartment sites. It constitutes barriers for the strangers to the neighborhood because the online maps do not provide a route option for apartment complexes’ internal streets. In this way, isolated apartment complexes are distributed in urban spaces to create effectively urban islands. The discontinuity of pedestrian paths in the city due to apartment complexes’ development directly and indirectly affects the physical and social network. Less connected pedestrian paths between apartment complexes and surrounding neighborhoods negatively affect people’s walking activities and increase the use of vehicles to bridge the disconnection of urban spaces, hindering social and physical sustainability.

Meanwhile, permeability is the core theory of new urbanism, which favors urban design based on the “traditional” (especially in the North American context) street grid. The new urbanist view also affects the policy of some governments. According to the UK’s *Street Traffic Guidance Manual*, the government encourages connected street networks by emphasizing that they encourage walking and biking and make places more comfortable to explore [7]. However, despite the criticism that such large-scale apartment complexes accelerate the separation of urban spaces, apartment complexes are still the preferred residential type. In particular, the construction of an apartment complex may renovate the old road network and improve the pedestrian environment through an increase in the road’s width and straightness. In addition, harmful effects of the existing apartment complex construction are mostly dealt with in the social aspect. In that case, it is necessary to objectively review the impact of the apartment complex construction on the physical environment of surrounding neighborhoods.

Therefore, this study aims to analyze the effect of the policy change to open apartment complexes on the pedestrian permeability and pedestrian accessibility and its influence on pedestrian efficiency of the city through these questions. The relative differences in accessibility measurements are considered as pedestrian efficiency (PE) in this study. This study also aims to determine whether there are better solutions to increasing accessibility by testing the new path planning inside apartment complexes using Voronoi diagrams. This paper explores how the spatial potential of different network configurations in the internal area of apartment complexes can generate better accessibility. The specific research questions are as follows. How do the spatial characteristics of the apartment complex affect the transmittance? How and to what extent does the road opening in the apartment complex change the apartment complex’s permeability and the surrounding area? Furthermore, how can the accessibility change by some design proposals to modify pedestrian networks inside apartment complexes using Voronoi diagrams?

## 2. Literature Review

Cities’ openness has been discussed by different urbanists. Modern urbanists claimed that their form of city is the best option for open city, because it offers more ground space for green or for solar [8]. However, planners such as Jane Jacobs claimed that openness is more related to social activity on the street; the city is open if social interaction is possible [9]. Therefore, Jacobs objects to modern planners such as Le Corbusier, since their concept is hindering the social activities and social interactions on street [9]. Social interaction increases in cities where walking is the chosen method of movement. Therefore, new urbanism suggests walkability as one of the key principles of a sustainable neighborhood [10]. In particular, walking has a positive influence on the formation of community consciousness in the neighborhood by inducing both movement through physical space and social and spatial interaction between people [11].

Unlike in the past, when there were many social and physical restrictions on movement, the development of transportation technology has enabled better movement options between spaces. However, walking is still a basic means of transportation that enables movement between two spaces while minimizing cost if physical strength permits. More significant, walking is a tool of ridership for accessing transit. With this point, the issue of accessibility becomes critical, because it determines whether people choose walking on the street. Meanwhile, accessibility measurements are used both for walkability studies and help to understand different aspects of an urban form such as its compactness, functionality, sustainability, equity, and centers of social interaction [12].

As an element of conflict caused by apartment complexes, there is an increase in walking distance due to the disconnection of urban pedestrian path. As a result of this closed form of the apartment complex, outside residents may bypass the apartment complex and try to access their destination using public transit. Briefly, the physical walking distance becomes longer, which acts as an obstacle to the purpose of passage through walking, which is one cause of reduction in walking activity [13,14]. Studies on how to integrate and increase the usefulness of the internal network of apartment complexes include Yang and Yu [15]. They use the Delanuay triangulation method to evaluate and compare average walking distances for currently used and potentially designed pedestrian networks for the apartment complexes in Seoul, South Korea. They present three strategies: transit-oriented development, complete street, and mobility enhancement. Similarly, Ai et al. [16] used Delanuay triangulation to test their experimental study on the spatial neighborhood relationship representation.

Improvement of the pedestrian environment promotes the walking activity of urban residents, which is likely to decrease the use of automobiles, thereby improving the urban environment. In particular, the improvement of pedestrian accessibility in neighborhoods due to the improvement of the pedestrian environment outside of the apartment complex affects the housing price [17,18]. Housing preference in areas with good public transportation and pedestrian access to school increases, so there is a positive correlation between pedestrian friendliness and housing prices [19,20]. There is also a statistically negative correlation between crime incidence rates and the walking activity occurrence on a certain street [21]. Jacobs’ theory of “eyes on the street” is another source that supports this negative correlation [9]. The excellent pedestrian environment in the neighborhood enhances walking accessibility to neighboring facilities and ensures pedestrian safety, so the willingness to live in the area increases [22]. Furthermore, the increase of citizens’ walking activities in external spaces due to pedestrian activation increases accessibility for the use of neighboring facilities in areas with excellent pedestrian environments. However, the construction of a large-scale apartment complex has both positive and negative aspects in terms of the pedestrian environment. Apartment sites negatively affect the walking experience for transit riders. This increases the length of the route, and the fences of gated communities create a tedious experience for pedestrians [23].

An early comprehensive study on neighborhood characteristics, street form, connectivity, and urban community identified spatial typologies and analyzed the patterns of growth, land use, and street layouts in the case study of the San Francisco Bay area [24]. Among the patterns, gridiron form allows the highest level of accessibility, and the form of lollipops on sticks (i.e., “cul-de-sacs”) has the lowest level of accessibility. However, high connectivity has a disadvantage of privacy, which decreases parallel to accessibility.

There are two main approaches to walkability in the literature: network connectivity and audit tools [25]. Briefly, connectivity analysis uses street networks to characterize the pattern of walkability, and audit tools help to document walkability from the perspective of pedestrians by using some standard forms. The former is more universal and can be easily adopted, whereas local characteristics may have different influence in the latter. In addition, the centrality concept has been used in walkability studies. Many of the centrality concepts were first developed in social network analysis [26]. However, the methods analyzing built environment and social capital are quite new. Liu et al. [27] investigated the street centrality and its impact on land use intensity in Wuhan, China. The authors suggested the walking network has a direct effect on activating walking. Similarly, Sun et al. [23] studied China’s policy to open gated communities to outsiders with a permeability perspective. The changes in betweenness analysis after opening the gated communities give some insights about how permeability of the city will affect the vitality of streets when there is a policy to un-gating apartment complexes. Yue et al. [28] also focused on the street centrality and its influence on urban vitality using social network review data in Wuhan, China. The straightness and betweenness measures have a direct positive effect on urban vitality. Instead of only main arteries, all streets become lively with a permeable neighborhood. In addition, better street centrality and accessibility directly affect housing values in neighborhoods [29].

There are some different approaches on the impact and benefits of opening policies. For instance, Dong et al. [30] approached the issue with a focus on its benefit for relieving the traffic burden. Their results showed that an opening policy will help decrease traffic congestion around gated community sites. Yang et al. [31] emphasized the benefit for the pedestrian and cyclist accessibility and route choices by measuring second-level scenarios of opening gated communities. However, to give a comprehensive answer to the benefits of opening the gated communities, additional analyses should be done in terms of accessibility changes by the strategies of opening the gated communities.

## 3. Materials and Methods

### 3.1. Study Area

**Figure 1:**
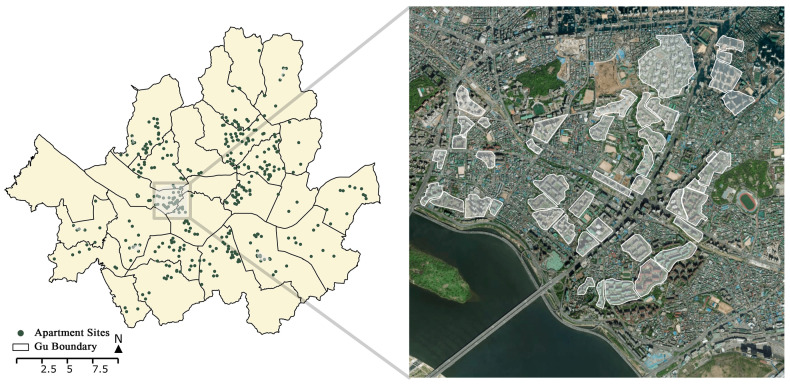
The case study area of Mapo-gu in Seoul and the clustering of apartment complex development sites.

**Figure 2:**
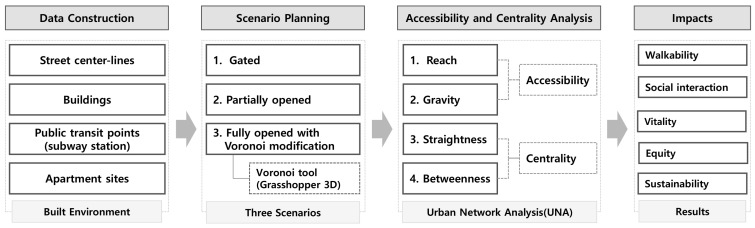
Conceptual framework.

The purpose of this study is to analyze based on the network connectivity the effect of the openness of an apartment complex on the pedestrian environment in a neighborhood. To this end, the impact of the road-opening policy in the apartment complex on the permeability of the pedestrian environment was compared using data on the progress of the 2018 Seoul Maintenance Project. The area of the east side of Mapo-gu, an apartment complex cluster, derived through analysis of k-mean clustering, was selected as the target area for analysis (Figure 1). Another reason to choose this area as the case is that the apartment complexes are distributed among the non-gated traditional neighborhoods (Figure 2). This would improve our understanding of how apartment complexes are affecting their surroundings. After determining the apartment complexes that are made of more than two blocks, each apartment complex was digitized by identifying the size of the development, the road network in the complex, the entrance, and the locational characteristics of facilities in the surrounding area using Google satellite images and Google Street View data. The total apartment sites analyzed in the Mapo-gu case area number 44, ranging from sites of three blocks to the sites of 40 blocks.

### 3.2. Data Sources

There are three main components of origin, network, and destination in this study. First, origin is the initial location of human a movement, which is usually considered to start from the place of residency. In our study, regardless of building type, we used the whole building stock in the case study area as origins. The building stock data used in this study were received from the source of “Road Name Address DB,” provided by the Ministry of Public Administration and Security. Second, the mean of transport has two different impacts for pedestrian activity; one of them is about walking through (betweenness and straightness measurement in our study are related to this) and the other is about walking to (gravity and reach measurements in our study are related to this). The data of network, which is the mean of transport, were generated based on street centerlines from Seoul road maps. Third, subway stations were chosen as the destination because subway stations are distributed in every part of city, and subways are the most used public transportation method in Seoul. According to the Korean Statistical Information Service’s Annual Modal Share in 2018, the share of subway on public transportation is 41%, whereas the share of bus is only 24% [4].

### 3.3. Methodology

One of the most common methodologies for network and accessibility studies is space syntax. There are various software and tools for space syntax methodology. The urban network analysis (UNA) framework has been used for this study. Contrary to deficiencies in space syntax and other network analysis methodologies, UNA has a useful modification in that it adds buildings (housing or other infrastructures) to representations, adopting a tripartite system that consists of three basic elements: edges, nodes, and buildings [32]. In the conception of this method, buildings are represented as a point, and each point is connected to the nearest street segment (edge) by the shortest line.

Figure 2 presents the conceptual framework. In this study’s measurements, two walking distances were considered: 400 m and 800 m. The first is the highest distance for comfortable walking, and the second is the highest distance level for acceptable walking. Studies suggest that up to 800–1000 m distance is acceptable for walking [31]. However, the most affordable walking distance, which is accepted as a standard by many authorities, is 400 m [33]. The indicators measured in this study include reach, gravity, straightness, and betweenness. Among these indicators, reach and gravity comprise the accessibility index, whereas betweenness and straightness comprise the centrality index. The higher the improvement in the values of these four measurements, the higher the flow of people on the street and the higher mobility and vitality along the streets.

The definitions of the measurements are as follows. First, “reach” measures the number of destinations that can be accessible in a certain radius area. Second, “gravity” measures the travel cost to access to destinations in a certain radius area. It can also be regarded as attractiveness, as being closer to the destination means higher attractiveness. Three, “straightness” measures how directly a route is being generated between origin and destination. Finally, “betweenness” measures to which degree a node is being used when a configuration of routes geo-located between all origin and destination.

**Figure 3:**
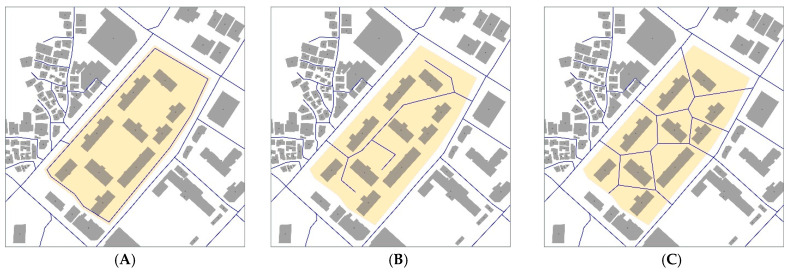
Three different scenarios of internal pedestrian network of apartment sites and their connection to surrounding neighborhoods: (**A**) Gated; (**B**) Partially opened; (**C**) Fully opened with Voronoi modification.

Three different scenarios of an internal pedestrian network of apartment sites and their connection to their surroundings are considered in the measurement: gated, partially opened, and fully opened with Voronoi modification (Figure 3). First, scenario 1 assumes that the internal network of apartment complexes is not used by outsiders (because it is gated or because the lack of awareness about the internal network of the site). Scenario 2 assumes that the internal network of apartment complexes is open to outsiders as it is (including small entrances, gates, and all pedestrian paths inside the complexes). Scenario 3 assumes the use of a Voronoi diagram to find an optimal network system to minimize the pedestrian walking time in accessing public transportation. In this hypothetical scenario, we re-design the entrances and internal network by a Voronoi diagram, considering the locations of buildings and outside street network.

The Voronoi diagram is defined as a cell formed by dividing the Euclidian line connecting the two most adjacent points by vertically dividing the lines. A Voronoi diagram consists of a Voronoi cell, the Voronoi space that surrounds a Voronoi cell, a Voronoi vertex, and a Voronoi foam [34]. Voronoi diagrams have been used widely for the delimitation of maritime zones and mapping coastal boundaries [35] and for mechanical engineering and robotics [36,37] as well as in urban planning and architecture for analyzing or designing indoor circulation, path planning, and service area studies [38,39,40,41,42]. In particular, the Voronoi diagram can describe the service area of the city network more accurately than can the traditional Euclidian network method [43]. This means that the space divided by a Voronoi diagram can simulate human walking activities more realistically.

**Figure 4:**
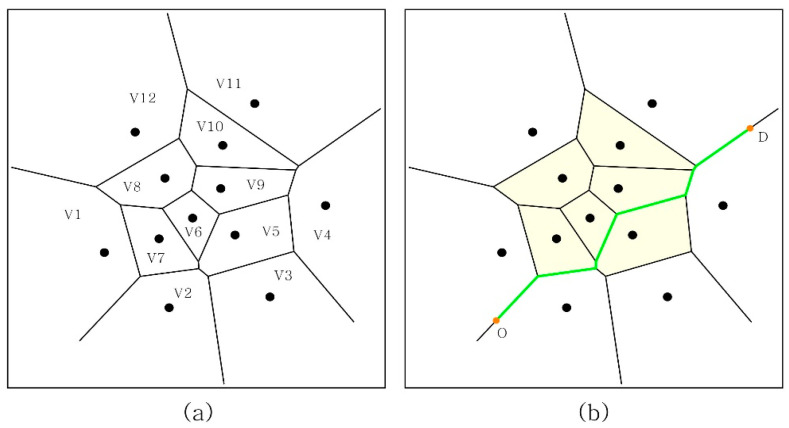
An example of environment created by Voronoi diagram using Grasshopper Voronoi tool: (**a**) V1-V12 (Voronoi Areas for each point); (**b**) Shortest connection of O and D on Voronoi boundaries. As an example, origin (O) is a building unit, and destination (D) is a subway station.

Additionally, in urban design, a Voronoi diagram gives the opportunity to draw the optimum shortest routes among the buildings or other obstacles of a built environment. Optimum shortest routes are different from geometrical shortest connections by not closely approaching obstacles among the route. Moreover, a Voronoi diagram helps to create environments for each building by not interrupting the others (Figure 4). To create a Voronoi diagram, we used the Voronoi plugin on Grasshopper3D visual programming language. Network studies have two elements in their models: nodes and routes. Thus, we define a network with our Voronoi diagram whose vertices are nodes and whose edges are routes.

### 3.4. Pedestrian Efficiency

In this study, the measurements of reach, gravity, straightness, and betweenness were considered as positive indicators for pedestrian environment. Therefore, the relative differences of these measurements in three scenarios were considered as pedestrian efficiency (PE). S1 stands for the measurement result of scenario 1 (gated). S2 indicates the measurement result of scenario 2 (partially opened). S3 means the measurement result of scenario 3 (fully opened with Voronoi modification). Meanwhile, PE1 indicates a pedestrian efficiency change for the hypothetical scenario 2 (partially opened with existing internal paths). PE2 means pedestrian efficiency for hypothetical scenario 3 (fully opened with modifying internal paths with Voronoi diagram). In addition, we examined the number of buildings with increasing accessibility and centrality in the case study area.

$$\mathrm{PE}1\left(\%\right)=\frac{S2-S1}{S1}\times 100,\;\mathrm{PE}2\left(\%\right)=\frac{S3-S1\;}{S1}\times 100$$

## 4. Analysis Results

The study focused on accessibility and centrality measurements of street networks by considering these measurements as an evaluation of pedestrian efficiency. Values of accessibility, namely reach and gravity, and centrality, namely straightness and betweenness, measurements to examine the spatial potential for pedestrian efficiency assessment have been calculated for three scenarios of pedestrian network: (1) the internal networks of apartment complexes are not used by outsiders; (2) the internal network of apartment complexes are open to outsiders with its existing entrances and path; and (3) the internal network of sites are opened and redesigned by the Voronoi diagram method, which generates the optimal shortest path. Accessibility and centrality measurements have been conducted for 13,405 buildings based on the destination of 10 subway stations in the case area.

In Table 1, the average values of reach, gravity, straightness, and betweenness of 13,405 buildings for radius area of 400 m and 800 m, and the percentages of PE1 (relative difference between scenario 1 and scenario 2) and PE2 (relative difference between scenario 1 and scenario 3) are listed. In addition, the number and percentage of buildings having accessibility and centrality gain in scenario 2 and scenario 3 compared to scenario 1 are added to the table. In general, we find that the average values of all reach, gravity, straightness, and betweenness, as well as the number of buildings having accessibility and centrality gain, respectively increase in scenarios 2 and 3. First, comparing scenarios 1 and 2, we find that the opening of internal network of complexes has a positive effect on all four indicators of reach, gravity, straightness, and betweenness, from 2.42 to 2.56% in a radius of 400 m and from 1.48 to 3.20% in a radius of 800 m. Furthermore, the values of four indicators of reach, gravity, straightness, and betweenness keep increasing in scenario 3, which is the scenario of redesigning a pedestrian path for optimum shortest distance by a Voronoi diagram, from 2.81 to 3.99% in a radius of 400 m and from 2.99 to 6.16% in a radius of 800 m.

The results clearly indicate that not only opening gated communities but also modifying their internal network with a Voronoi diagram will increase all the accessibility and centrality values. Among the indicators, straightness has the highest percentages of change in three configurations, especially in a radius area of 800 m, as it increases up to 6.16% in scenario 3. It can be explained as that apartment complexes are hindering direct access to facilities by their gated conditions. Particularly, this is more visible in the case of longer destinations than shorter ones.

We analyze whole buildings in the case area, which includes 13,405 buildings. To understand how many of the 13,405 buildings in the case area have accessibility and centrality benefiting from open gates and redesigning pedestrian path, we calculated the number and percentage of buildings having accessibility and centrality gains in scenarios 2 and 3 by considering scenario 1 as the base. The number of buildings benefiting from such an opening policy increases drastically in all four measurements. As an example, for the radius area of 800 m in scenario 3 (fully opened with Voronoi modification), the number of buildings benefitting increases up as following: 830 units (6.19%) in reach, 2645 units (19.73%) in gravity, 2657 units (19.82%) in straightness, and 2063 units (15.39%) in betweenness.

**Figure 5:**
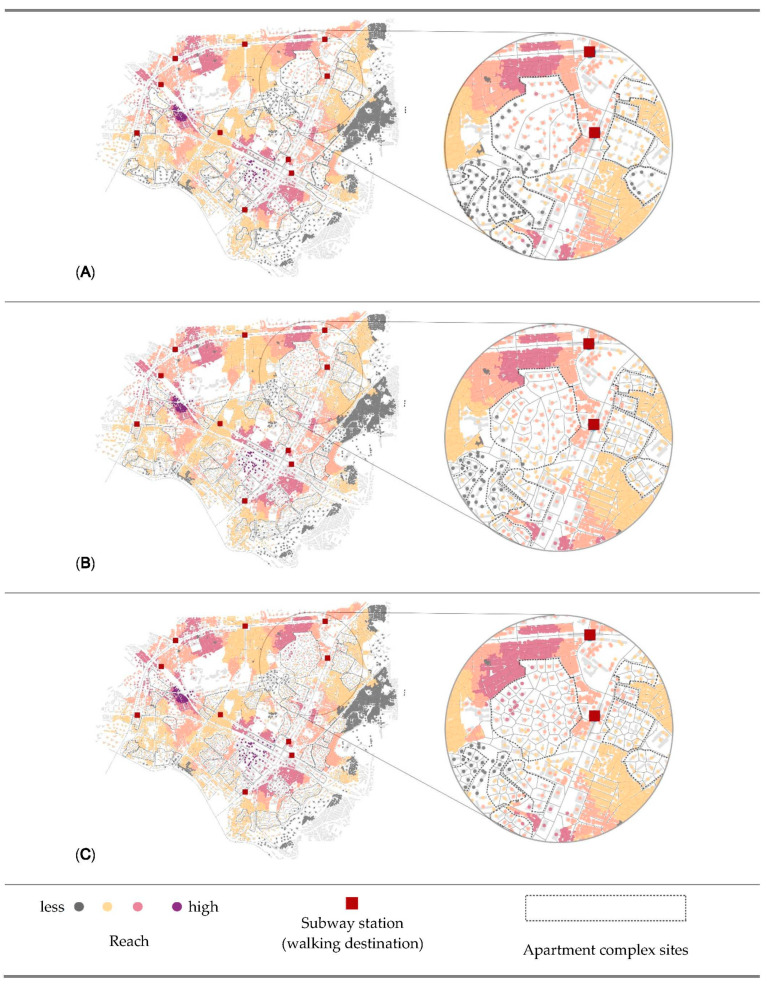
Visualization of Reach results that present the spatial accessibility of the cases in a radius of 800 m. (**A**) Scenario 1—Gated, (**B**) Scenario 2—Partially opened, (**C**) Scenario 3—Fully opened with Voronoi modification.

**Figure 6:**
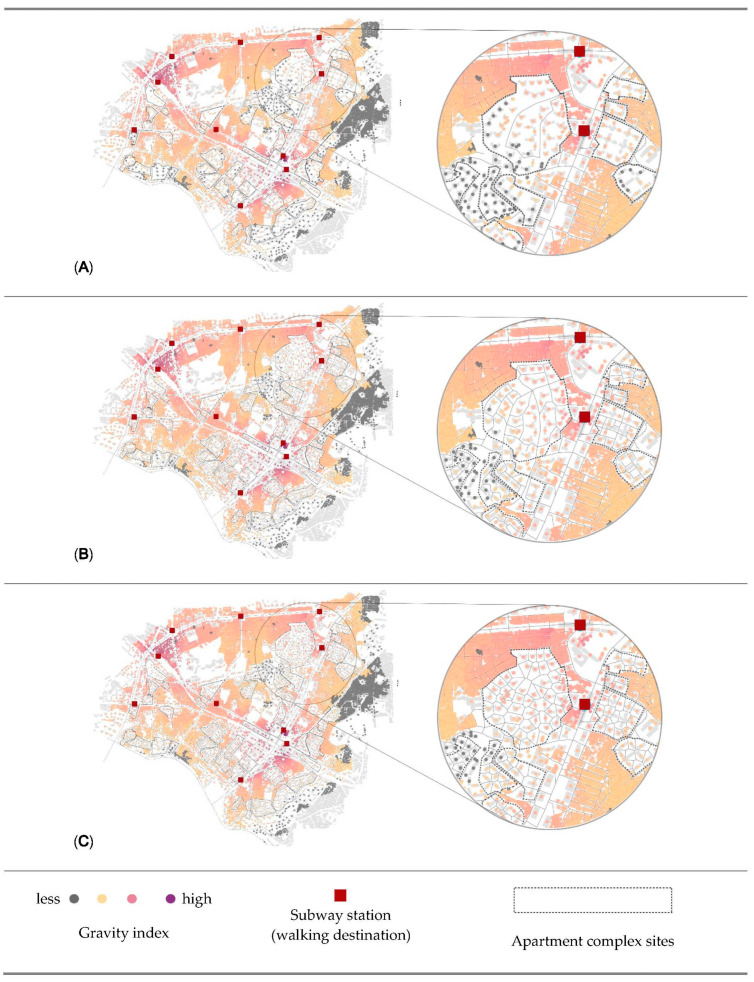
Visualization of Gravity results that present the spatial accessibility of the cases in a radius of 800 m using beta value of 0.00217. (**A**) Scenario 1—Gated, (**B**) Scenario 2—Partially opened, (**C**) Scenario 3—Fully opened with Voronoi modification.

**Figure 7:**
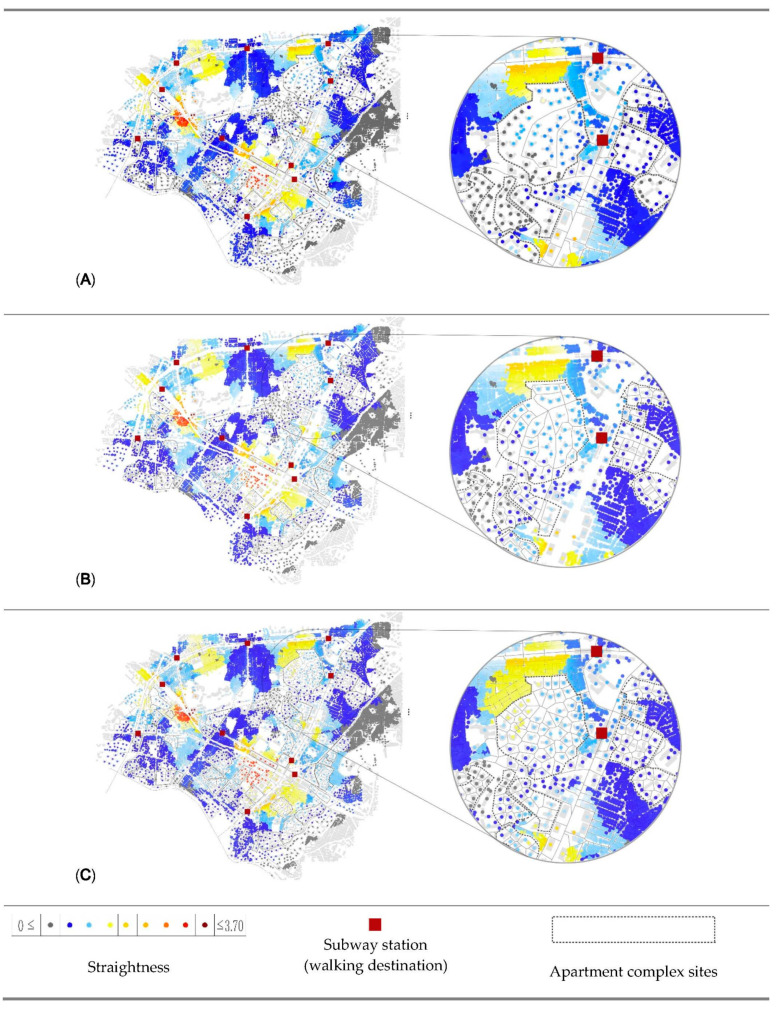
Visualization of Straightness results that present the measurement of direct spatial accessibility of the cases in a radius of 800 m. (**A**) Scenario 1—Gated, (**B**) Scenario 2—Partially opened, (**C**) Scenario 3—Fully opened with Voronoi modification.

Figure 5, Figure 6 and Figure 7 visually map the reach, gravity, and straightness measurements for three scenarios. First, in the visualization of reach (Figure 5) in dark pink indicates the highest reach value, followed by light pink and brown. Gray shows the lowest reach measurements. Buildings located between more than one destination in a short distance have the highest value of reach. Second, in the visualization of gravity (Figure 6), dark pink is the indicator of high accessibility level, while gray indicates less accessible origins. Different from the reach measurement, buildings that are the closest to any of destination mostly have the highest values of gravity. Last (see Figure 7), dark brown indicates the highest straightness value, following yellow and light blue, and dark blue shows the lowest straightness measurements. Gray indicates the origins beyond 800 m from destinations.

According to the results, the street pattern generated by the Voronoi diagram provides the best accessibility result. In addition, it is one of the best options for walkability, because it has smaller building unit and more intersections. As mentioned in the literature review, small units and more interactions provide walkers direct travel and more route choices. The visual results in Figure 5, Figure 6 and Figure 7 also indicate that the benefits of accessibility and centrality by opening policies are not only for the buildings outside of apartment complexes but also buildings inside complexes. Several points inside the complexes are turning brown or pink from gray in scenarios 2 and 3. Briefly, the results of analysis proved our hypothesis that the opening policies of apartment complexes would help the improvement in accessibility and centrality measurements. Pedestrians can reach their destinations in a shorter time, and the vitality of streets should increase by the increasing centrality of a neighborhood and the permeability of a city. Furthermore, it also shows that better design proposals can create shorter distances for pedestrian networks.

## 5. Conclusions

Pedestrian accessibility to public transit is essential for increasing public transit ridership in general and walkability in particular. Improved accessibility influences the local and regional economics as well as social health of urban communities. This study analyzed the effect of the closed-ness and open-ness of apartment complexes on the connectivity of urban spaces. The change in accessibility of public transport facilities in the surrounding area of apartment complexes was spatially visualized using three scenarios. First, the internal networks of apartment complexes are not used by outsiders. Second, the internal networks of apartment complexes are open to outsiders with existing entrances and paths. Third, the internal network of sites is opened and redesigned by a Voronoi diagram method generating the shortest optimal path.

The analysis confirmed that the policy of opening the pedestrian path from the neighborhood around the complex to the interior of the apartment complex improved the accessibility and centrality of the network including the apartment complex, thereby increasing the accessibility to public transportation facilities. Therefore, to secure public access to urban spaces in the future development of large-scale apartment complexes and to increase equity in the use of public transportation, this study presented the basis for introducing an open policy of apartment complexes. In addition, the results of this study can help urban policy makers and urban planners to increase the sustainability of their neighborhoods by creating a spatial layout to enhance urban spaces and providing a pedestrian-centered urban space rather than one that is car-centered.

The pattern of streets has a strong impact on the quality of the urban environment. The spatial pattern of urban streets directly affects connectivity and accessibility as well as privacy and safety. As this study shows, accessibility can be improved significantly with different configurations. However, it would be incomplete to evaluate the issue of walkability only in the context of accessibility based on street network patterns. The spatial pattern of streets may affect opposite impacts. For instance, increasing accessibility may mean decreasing privacy and safety. Therefore, urban designers and policy makers need to devise a legible street pattern that provides pedestrian, bicycle, and transit access without sacrificing privacy and safety. In addition, this study focused on the issue of accessibility to subway stations, which is one of the most important amenities. However, there are other essential facilities of commercial, education, medical, parks, and public services in the neighborhoods. Finally, the problem may not be only the closed-ness of apartment complexes by a fence or wall. It needs to be studied whether superblock-centered urbanism is good for pedestrianism and lively streets. The opening of gated community may not be enough to make streets lively unless they are designed organically with the surrounding urban fabric. Hence, the opening of gated communities may not be the only solution; we may also need to quantify the impact of superblock-centered urbanism on the quality of life in the surrounding neighborhoods.

## Figures and Tables

**Table 1:** Analysis results for three scenarios.

Measurement		Radius	Scenario 1 (Gated)	Scenario 2 (Partially Opened)	Scenario 3 (Fully Opened with Voronoi)	Pedestrian Efficiency (%)	
						Scenario 2	Scenario 3
Reach	No. of benefitting building *	400 m	-	113	171	0.84%	1.28%
		800 m	-	464	830	3.46%	6.19%
	Average index value	400 m	0.3549	0.3635	0.3678	2.42%	3.61%
		800 m	1.4071	1.4432	1.4759	2.57%	4.89%
Gravity	No. of benefitting building *	400 m	-	174	356	1.30%	2.66%
		800 m	-	1645	2645	12.27%	19.73%
	Average index value	400 m	0.1989	0.2037	0.2059	2.44%	3.51%
		800 m	0.4814	0.4940	0.5035	2.62%	4.59%
Straightness	No. of benefitting building *	400 m	-	171	359	1.28%	2.68%
		800 m	-	1653	2657	12.33%	19.82%
	Average index value	400 m	0.2628	0.2695	0.2733	2.54%	3.99%
		800 m	1.0707	1.1050	1.1366	3.20%	6.16%
Betweenness	No. of benefitting building *	400 m	-	357	491	2.66%	3.66%
		800 m	-	1416	2063	10.56%	15.39%
	Average index value	400 m	5.5168	5.6582	5.6718	2.56%	2.81%
		800 m	38.4715	39.0389	39.6230	1.48%	2.99%

* The number of benefitting buildings are all buildings that have accessibility or centrality gain under scenario 2 or 3 compared to scenario 1 of a gated community. The total number of buildings in the case study area is 13,405. For instance, pedestrian efficiency (0.84%) of reach index for scenario 2 was calculated by 114 divided by 13,405.

## Data Availability

Not applicable.
